# Pathway analysis of genome-wide data improves warfarin dose prediction

**DOI:** 10.1186/1471-2164-14-S3-S11

**Published:** 2013-05-28

**Authors:** Roxana Daneshjou, Nicholas P Tatonetti, Konrad J Karczewski, Hersh Sagreiya, Stephane Bourgeois, Katarzyna Drozda, James K Burmester, Tatsuhiko Tsunoda, Yusuke Nakamura, Michiaki Kubo, Matthew Tector, Nita A Limdi, Larisa H Cavallari, Minoli Perera, Julie A Johnson, Teri E Klein, Russ B Altman

**Affiliations:** 1 Department of Genetics, Stanford University School of Medicine, Stanford, CA 94305, USA; 2 Department of Biomedical Informatics, Columbia University, New York, NY 10032, USA; 3 Biomedical Informatics Training Program, Stanford University School of Medicine, Stanford, CA 94305, USA; 4 Wellcome Trust Sanger Institute, Hinxton, UK; 5 University of Illinois at Chicago, Department of Pharmacy Practice, Chicago, IL 60612, USA; 6 Clinical Research Center, Marshfield Clinic Research Foundation, Marshfield, WI 54449, USA; 7 Research Group for Medical Informatics, Center for Genomic Medicine, RIKEN, Tokyo, Japan; 8 Aurora St. Luke's Medical Center, Milwaukee, WI, USA; 9 Department of Neurology, University of Alabama at Birmingham, AL 35294, USA; 10 University of Chicago, Department of Medicine, Chicago, IL 60637, USA; 11 University of Florida, Department of Pharmacotherapy and Translational Research, Gainsville, FL 32610, USA; 12 Department of Bioengineering, Stanford University School of Medicine, Stanford, CA 94305, USA

## Abstract

**Background:**

Many genome-wide association studies focus on associating single loci with target phenotypes. However, in the setting of rare variation, accumulating sufficient samples to assess these associations can be difficult. Moreover, multiple variations in a gene or a set of genes within a pathway may all contribute to the phenotype, suggesting that the aggregation of variations found over the gene or pathway may be useful for improving the power to detect associations.

**Results:**

Here, we present a method for aggregating single nucleotide polymorphisms (SNPs) along biologically relevant pathways in order to seek genetic associations with phenotypes. Our method uses all available genetic variants and does not remove those in linkage disequilibrium (LD). Instead, it uses a novel SNP weighting scheme to down-weight the contributions of correlated SNPs. We apply our method to three cohorts of patients taking warfarin: two European descent cohorts and an African American cohort. Although the clinical covariates and key pharmacogenetic loci for warfarin have been characterized, our association metric identifies a significant association with mutations distributed throughout the pathway of warfarin metabolism. We improve dose prediction after using all known clinical covariates and pharmacogenetic variants in VKORC1 and CYP2C9. In particular, we find that at least 1% of the missing heritability in warfarin dose may be due to the aggregated effects of variations in the warfarin metabolic pathway, even though the SNPs do not individually show a significant association.

**Conclusions:**

Our method allows researchers to study aggregative SNP effects in an unbiased manner by not preselecting SNPs. It retains all the available information by accounting for LD-structure through weighting, which eliminates the need for LD pruning.

## 

Accurate prediction of clinical phenotypes, such as disease manifestations or drug therapeutic response, using genotype data is a fundamental challenge in translational biology. A primary method for establishing phenotype-genotype links is through Genome-Wide Association Studies (GWAS), which interrogate the genome in regions of common variability and attempt to statistically associate this variability to the phenotype of interest [1]. This phenotype may be a disease trait, or in the case of a pharmacogenetic GWAS, a drug-response phenotype such as the drug efficacy, adverse events, or required dose.

Warfarin, an anticoagulant prescribed to over 30 million patients annually to prevent thromboembolic events, is a drug with known pharmacogenetic influences [2-4]. The dose of warfarin required for an adequate level of anticoagulation cannot reliably be predicted from clinical factors alone. Patients on warfarin may require doses ranging from less than 10 mg/week to over 100 mg/week in order to achieve the same level of therapeutic anticoagulation [2,3]. Determining the therapeutic dose is critical because overdosing can lead to adverse events such as bleeding while underdosing leaves patients at risk for thromboembolic events [5]. Genetic variations in both pharmacokinetic and pharmacodynamic genes relevant to warfarin impact the required dose [2]. The most important variants influencing warfarin dose occur in genes encoding warfarin's target, VKORC1, and a major metabolizing enzyme, CYP2C9 [6-8]. Dosing algorithms using key variants from these genes along with clinical factors can predict stable warfarin dose to some extent, and have the potential to help avoid the adverse events caused by prolonged "guess-and-test" dosing [2,9-11]. Approximately 30% of the variability in warfarin dose requirement is explained by the genetics of VKORC1 and CYP2C9. However, current dosing algorithms fail to fully explain the variation in dose, even when using clinical factors in addition to the genetic information. These algorithms perform particularly poorly in African Americans, whose decreased variation in VKORC1 and CYP2C9 hinders dose prediction algorithms based on variation present in European and Asian populations [12-14].

The biological pathways involving warfarin are well characterized, and warfarin metabolism genes show high genetic variability not only between individuals but also between populations, making it cumbersome to refine the warfarin dosing equations for each specific population [14-16]. Furthermore, rare variants in important genes may have crucial effects that cannot be quantified by traditional genome-wide association methods [17]. Aggregating information from multiple single nucleotide polymorphisms (SNPs), that individually do not show significant association with phenotypes of interest can improve our power to detect important variation. For example, we have shown that rare variants of CYP2C9 can be pooled to create an indicator variable that significantly improves warfarin dose prediction [18]. SNPs can be grouped together in a data-driven manner using the strength of association or by analyzing previously characterized genes and pathways. Data-driven methods select the most promising (nearly significant) SNPs and combine the information from these SNPs to create a summary statistic that can be associated with the phenotype of interest. In this setting, machine learning approaches, including random forest regression, boosted regression tree, and support vector regression, can be used to select the subset of SNPs and clinical covariates for predicting phenotypes [19,20]. However, these data-driven methods are at risk of overfitting the data, producing a model not generalizable to other datasets or populations [20,21]. Moreover, statistical learning methods can be biased by LD structure and minor allele frequencies, requiring additional pre-processing [22].

Biologically-driven methods use information from known pathways to group SNPs together, with the assumption that the SNPs may have similar effects on phenotype and thus can be aggregated. Such methods allow for the combination of SNPs that may not be significant alone, but in aggregate, are associated with pathway dysfunction. Biologically-driven pathway-based methods have been used for analyzing gene expression data, proteomic data, and are now emerging as a mode of analysis for genotyping and sequencing data [23][24].

Pathway-based methods group mutations from genes in a biologically relevant pathway [24]. For instance, Baranzini et al. combined nominally-significant SNPs (with p< 0.05) in a MS GWAS to create gene groupings and then compared these groupings to characterized interaction networks to find sub-networks that were enriched for MS genes. They identified putative pathogenesis pathways that were consistent with the biological systems thought to be important in Multiple Sclerosis [25]. Similarly, a pathway analysis of the Wellcome Trust Case Control Consortium (WTCCC) looked at the seven disease GWAS and used the most statistically significant SNP in each gene in order to create a "score" metric for that gene. Using the top genes, Torkamani et al. looked for pathways that were enriched for the gene-set [26]. A pathway-based approach of studying the pharmacogenomics of purine and pyrimidine antimetabolites in gene expression data revealed that analyzing data using gene-sets rather than a single gene approach was able to detect important groups of genes that impact drug cytotoxicity [27].

These studies demonstrate the potential value of pathway-based methods and underscore the importance of aggregated genetic effects. However, they have focused primarily on gene expression data and can be limited by a SNP selection strategy that relies on individually significant p-values from a single-locus analysis. Here, we use biologically curated pathways, such as warfarin metabolism, to create a statistic that can be tested for association with warfarin dose. In particular, we aggregate the mutational burden of genes in the metabolic pathway of warfarin and demonstrate that this mutational burden explains some of the missing heritability in warfarin dose.

Several methods exist for aggregating SNPs across genes or pathways. VAAST assigns gene scores using both variant frequency data and amino acid substitution data to test the association of entire genes to cases and controls [28]. The weighted-sum method also combines rare variants and compares the burden of mutation between cases and controls to determine genes or pathways of interest [29]. In addition, we have used genotype frequency to aggregate SNPs across a gene [30]. All of these methods are designed to work best when low frequency variants contribute greatly to gene scores [28,29]. Moreover, linkage disequilibrium (LD) confounds the ability to aggregate variants since multiple variants may be effectively carrying the same "information" and including them together may amount to double counting and over-estimation of association strength. Some methods use permutation testing in order to address this; however, permutation testing does not properly account for LD since it considers correlated variables to be independent. The alternative is to remove SNPs that are in LD above an arbitrary threshold cutoff, but this approach leads to a significant loss of information [31]. Our method accounts for LD structure by using a tree-based weighting scheme initially developed in the context of sequence analysis [32].

## Methods

### Data sources

We used three data sets from prior studies: all subjects were on stable doses of warfarin with repeated INR measurements between 2-3.

1. Genotypes for 188 patients of European descent with a stable warfarin dose and clinical covariates were obtained from Cooper et al [33]. Samples were genotyped using the Illumina HumanHap 550k, version 3 BeadChip (Illumina, San Diego, CA). SNPs with minor alleles in less than 4 samples (1.3% allele frequency) were excluded. Average call rate for SNPs was 98.8% [33].

2. We obtained genotypes from 233 venous-thromboembolism patients of European descent on warfarin from the Malmo Thrombophilia Study [34]. These subjects were genotyped on the Illumina 670 chip (Illumina, San Diego, CA). SNPs with a minor allele frequency (MAF) below 1%, MAF between 1 and 3% and call rate under 99%, and MAF over 3% and call rate below 98% were excluded. All included samples had a call rate above 95%. Ethnic outliers were removed using principle component analysis with Hapmap3 samples.

3. We obtained genotypes from 342 African Americans (the IWPC GWAS) from different IWPC sites. Subjects were genotyped using the Illumina 610 Quad BeadChip at the RIKEN Center for Genomic Medicine in Yokohama, Japan. SNPs with less than 2% minor allele frequency were excluded except for SNPs in VKORC1 and CYP2C9, because of evidence suggesting their possible involvement in warfarin dosing. Data collection and quality control were based on previously described IWPC protocols [2,35-37].

For each dataset, PCA was performed on the genotype data using the pca_module package (version 1.1.02) for Python (version 2.7.1).

### Constructing a pathway score: gene aggregation, LD weighting methods, and pathway mapping

**Figure 1:**
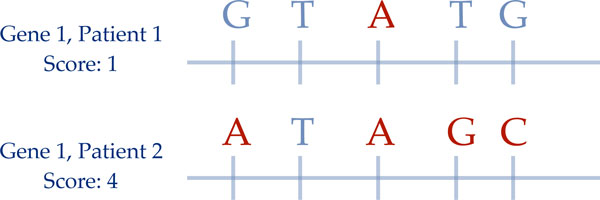
**Aggregate number of minor alleles (A#m) scoring gives each gene 1 point for every minor allele, and then allows us to compare the phenotype to the scores across the warfarin metabolic pathway**.

Aggregate Number of Minor Alleles (A#m) sums the number of minor alleles observed in a given gene (with each individual position having a maximum score of 2). It assigns a point to the gene whenever a SNP is the minor allele, or two if the SNP is homozygous for the minor allele (Figure 1). Major alleles are derived from the control population and whenever any allele that is not a major allele appears, a point is given to the gene's score. In the case of no control population, (e.g. both high drug dose and low drug dose populations), the major alleles are derived across the entire population. We obtained the major alleles using plink (version 1.07) over the entire population of each individual dataset (e.g. Cooper et. al. for Cooper major alleles).

**Figure 2:**
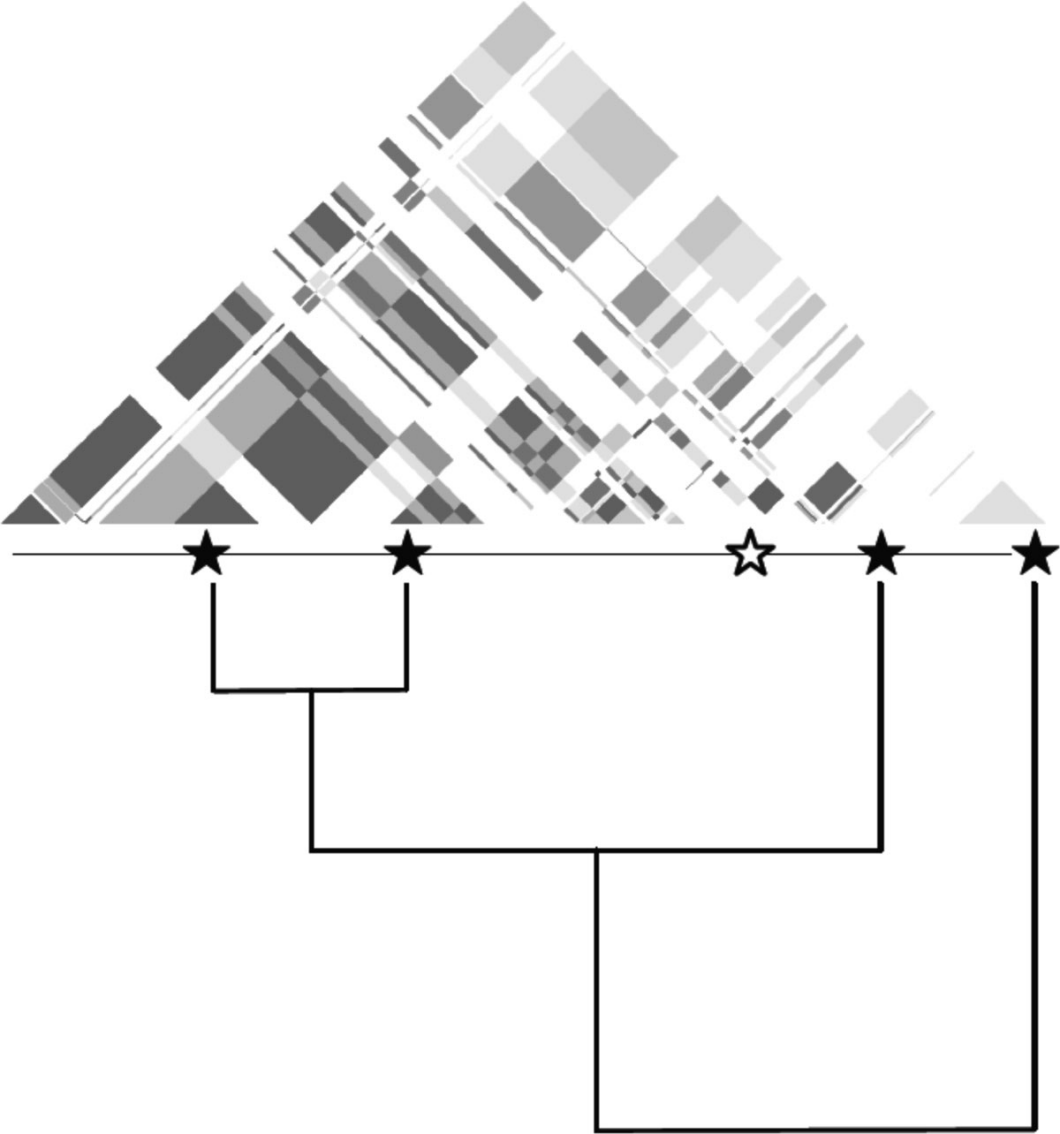
**LD was calculated between SNPs within 1000 kb in the pathway**. SNPs were clustered based on the LD r^2^ values. We calculated the weights of each leaf node (SNP) using the Gerstein-Sonnhammer-Chothia algorithm.

**Figure 3:**
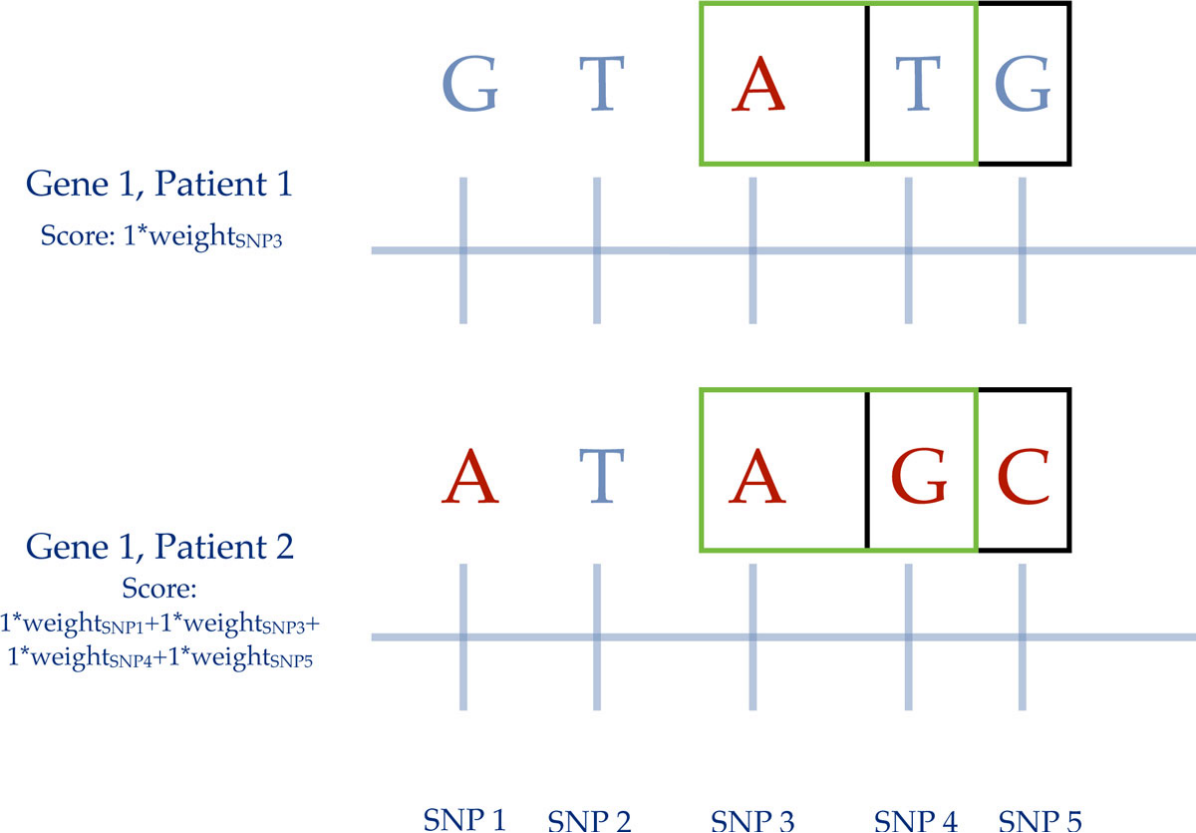
**LD-weighted A#m scores are calculated by taking the sum of all minor alleles multiplied by their respective weights**. In this case, the SNPs in the black box are in tight LD, while the SNPs in the green box are in moderate LD. With SNP weighting, rather than pruning the SNPs in LD, SNPs are weighted based on the contribution of independent information to the A#m score.

To account for LD, we used an algorithm previously developed for protein sequence similarity to weight a SNP based on its LD with nearby SNPs [32]. We clustered SNPs using the LD r^2 ^as the similarity metric. SNPs were clustered using single linkage clustering (where the shortest distance between any two leaves of a cluster is used as the cluster difference). The resulting clusters were used to create a bifurcating tree that relates all loci using LD (Figure 2). LD (r^2^) for each SNP was calculated for all pathway SNPs within 1 Mb using plink (version 1.07). Once clustered, we used the Gerstein-Sonnhammer-Chothia algorithm to compute the weights of each leaf node (SNP) [32]. This algorithm downweights overrepresented sequences; in this case, SNPs in LD get downweighted because they carry similar information. The LD-weighted score of each pathway was determined by summing the LD-weight of each minor allele in each given SNP (Figure 3).

We mapped SNPs to genes using dbSNP based on build hg19 of the human genome. For NCBI's hg19, we used the default feature set (introns, exons) of all genes and added 10,000 bases upstream (5') and 3,000 bases downstream (3') in order to capture regulatory regions.

We determined drug metabolic pathway by selecting the metabolic enzymes in PharmGKB's warfarin pharmacokinetic pathway (PA145011113) and the drug target pathway by using PharmGKB's warfarin pharmacodynamic pathway (PA145011114), available for download at http://pharmgkb.org[38,39].

### Pathway and weighting method validation and replication

We performed several tests using our pathway method to assess how variation in warfarin metabolizing enzymes influenced dose prediction and the effectiveness of our weighted pathway metric. First, we applied our pathway method in a cohort of European descent to test the significance of pathway-level variation in predicting dose. Next, we dissected the pathway score into two pieces -- CYP2C9 and the rest of the pathway and tested how these two separate variables predicted dose. This helped determine whether or not the pathway was being driven by CYP2C9, which is known to contain many important warfarin variants [2,8]. Then we tested whether our biologically derived warfarin metabolic pathway was empirically significant through 100 permutations of random biological pathways using the Cooper et al. data. We then predicted dose in another cohort of European ancestry using all available covariates and the pathway score. To show broad population applicability, we applied our pathway score to predict dose in an African American cohort. We evaluated clinical utility, by using the pathway score as a variable for the warfarin pharmacogenetic equation in the African American cohort (the only cohort with all the pharmacogenetic equation variables available) [2]. Finally, to demonstrate the generalizability of our weighted pathway method, we compared weighting SNPs based on LD vs. pruning SNPs at various LD cutoffs.

### Pathway method validation in Cooper et al. cohort

We produced the A#m score for the warfarin metabolic and pharmacodynamic pathway in a population of 188 patients (182 of European descent and 6 of Hispanic descent) on warfarin. We used a linear regression to model warfarin dose with the warfarin metabolic pathway A#m and known clinical and genetic covariates: age, weight, race (principal components 1 and 2 of the genotype data), amiodarone, losartan, VKORC1 (rs9923231) and CYP2C9 status (carrier or homozygote for *3; *2 variant was not genotyped and could not be imputed from the available data). We excluded nineteen subjects who were missing one of the above covariates. We used an ANOVA to test the difference between the models including and not including the pathway score. All statistical analyses were performed using the R statistical package (version 2.10.1).

### Pathway method validation in Cooper et al. cohort without CYP2C9

We removed CYP2C9 scores from the pathway score in the Cooper et al. data. Then, we modeled warfarin dose using a linear regression with the aforementioned clinical and genetic covariates and individually added either the CYP2C9 A#m score or warfarin metabolic A#m score without CYP2C9 as covariates. All statistical analyses were performed using the R statistical package (version 2.10.1).

### Post hoc evaluation of 100 permutations of random biological pathways in the Cooper et al. data

We ran 100 permutations of random pathways from a combination of the PharmGKB, Biocarta, and Pathway Interaction Database using MySQL (version 5.1.14). We calculated the score for each random pathway using the A#m method previously described using the Cooper et al. data. We then modeled warfarin dose using a linear model with the previously mentioned genetic and clinical covariates and each pathway. We used the random pathways to compute a background distribution of correlations. The empirical p-values were then estimated from this distribution, and reported in addition to traditional statistical tests.

### Pathway method replication in IWPC Malmo cohort

We also applied pathway scores in another cohort of 233 individuals of European descent genotyped through the International Warfarin Consortium (IWPC). For the covariates, we used age, height, weight, principle component 1, principle component 2, rs1799853 (CYP2C9 *2), rs1057910 (CYP2C9 *3), rs9923231 (VKORC1 -1639 G>A), and rs9934438 (VKORC1 1173 C>T). The VKORC1 SNPs were in perfect LD, and thus, only one was used in the regression. We excluded ten subjects because they were missing some of the above covariates. Amiodarone and aspirin status were not available.

### Pathway method replication in IWPC African American GWAS cohort

We replicated this method in a population of African Americans genotyped by the International Warfarin Pharmacogenetic Consortium (IWPC). We used a similar set of covariates as used by the IWPC warfarin pharmacogenetic equation with the addition of our pathway scoring metric [2]. These covariates were age, height, weight, race (principal components 1 and 2 of the genotyping data as ancestry), use of aspirin, amiodarone, rs1799853 (CYP2C9 *2), rs1057910 (CYP2C9 *3), rs9923231 (VKORC1 -1639 G>A), and rs9934438 (VKORC1 1173 C>T). The VKORC1 SNPs were in perfect LD, and thus, only one was used in the regression. None of the patients were on enzyme inducers (carbamazepine, rifampin, phenytoin). We excluded 40 subjects that were missing one of the above covariates.

### Metabolic pathway score as a covariate in the IWPC dosing equation in the IWPC African American GWAS Cohort

We calculated the predicted dose of each individual in the IWPC African American cohort using the IWPC dose equation [2]. We then calculated the residual dose by subtracting the IWPC predicted dose from the actual dose. We used a linear regression to model the residual dose with the A#m pathway score. The Cooper et al. and IWPC Malmo cohorts were missing key covariate information necessary for the IWPC pharmacogenetic equation, and thus, we were unable to calculate an IWPC predicted dose for these groups.

### Weighting method validation in Cooper et al. data and IWPC African American GWAS with LD-pruning

We LD-pruned the Cooper et. al. pathway SNPs and the IWPC African American GWAS pathway SNPs at incremental r^2 ^cutoffs from 0.1 to 0.9. Variants were pruned using plink v1.07 with pairwise genotypic correlations using the default window size of 50 SNPs and a sliding window of 5 SNPs.

We used the resulting sets of pruned SNPs to generate A#m scores without weighting. We modeled warfarin dose using a linear regression with the aforementioned clinical and genetic covariates with the A#m scores generated at the different pruning r^2 ^cutoffs.

## Results

### Known metabolic pathway aggregation improves dose prediction in 169 individuals of European descent

The Cooper et al. cohort included 188 patients (182 of European descent and 6 of Hispanic descent) on warfarin doses ranging from 1 mg/day to 15.54 mg/day. A summary of the patient demographic data of the Cooper et al. cohort is available in Table 1. Nineteen observations were deleted due to missing data, and a comparison of demographic data between included and excluded data is in Additional file 1 Table S1.

**Table 1:** Cooper et al.

Covariate	Mean/% Patients	Standard Deviation	Coefficient	p-value
Age	58.7	15.7	-0.0407	6.43E-05*

Weight (lbs)	195.3	45.3	0.0109	1.32E-03*

Amiodarone	14.3%	-	-1.393	1.11E-03*

Losartan	9.3%	-	-0.420	4.14E-01

VKORC1 AG	46.6%	-	2.157	1.03E-05*

VKORC1 GG	41.0%	-	3.821	2.78E-13*

CYP2C9 status	29.2%	-	-1.330	1.77E-04*

Principal Component 1	-	38.8	-0.000389	9.19E-01

Principal Component 2	-	25.5	-0.00576	3.15E-01

A#m Metabolic Pathway	19.38	3.57	-0.0999	2.44E-02*

patient data, regression coefficients, and p-values

The A#m metabolic pathway consisted of 49 SNPs in 7 genes (Additional file 1 Table S2), and the A#m score distribution can be found in Figure S1. We found that the A#m metabolic pathway score was a significant predictor of the stable warfarin dose (F = 5.1708, p = 0.0244) (Table 1). We had an improvement of 1.4% in adjusted r^2 ^with the addition of the weighted pathway scores (0.466 to 0.48). The A#m target (pharmacodynamic) pathway was not a statistically significant predictor of warfarin dose and was not tested in the subsequent cohorts.

### Metabolic pathway score is not driven by CYP2C9 score

When CYP2C9 scores were calculated separately from the metabolic pathway and both were used as covariates, neither CYP2C9 scores nor pathway scores were statistically significant, though the A#m pathway scores without CYP2C9 was marginal (p = 0.0681) (Additional file 1 Table S3). However, the full A#m pathway score is statistically significant (p = 0.0244, Table 1). This significance was further confirmed by 100 permutations of random pathways, which we used to calculate an empirical p-value cut-off of 0.07 for significance.

### Metabolic pathway score marginally significant in replication cohort of 223 individuals of European descent

The IWPC Malmo cohort included 233 individuals of European descent on warfarin doses ranging from 1.25 to 15.14 mg/day. Additional demographic information related to warfarin dose covariates can be found in Additional file 1 Table S4. Ten observations were excluded due to missing data. For this cohort, the A#m pathway consisted of 84 SNPs in 7 genes (Additional file 1 Table S5), and the A#m score distribution can be found in Figure S2. The A#m pathway score was marginally significant (F = 3.30, p = 0.0710); coefficients and p-values for each covariate can be found in Additional file 1 Table S4.

### Metabolic pathway score improves dose prediction in a cohort of 302 African Americans

We replicated this method in a population of African Americans genotyped by the International Warfarin Pharmacogenetic Consortium (IWPC). This population consisted of 342 individuals on doses of warfarin ranging from 1.43 to 17.41 mg/day. Patient demographic data is summarized in Table 2. The metabolic pathway was created using 122 available SNPs in 7 genes (Additional file 1 Table S6), and the A#m score distribution can be found in Additional file 1 Figure S3. With the IWPC African Americans, 40 observations were not used in the analysis due to missing data; a comparison of covariates between included and excluded data can be found in Additional file 1 Table S7. Dose was not significantly different between individuals included in the analysis and individuals removed due to missing data.

**Table 2:** IWPC African American GWAS Cohort patient data, regression coefficients, p-values

Covariate	Mean/% Patients	Standard Deviation	Coefficient	p-value
Age	56.81	14.66	-0.350	1.32E-07

Weight (kg)	94.55	28.07	0.136	1.27E-04

Height (cm)	171.7	10.642	0.243	0.0105

Amiodarone	4.68%	-	-13.155	2.02E-03

Aspirin	27.49%	-	-7.21	9.58E-04

VKORC1 rs9923231 GG	82.16%	-	-	-

VKORC1 rs9923231 AG	16.96%	-	-7.422	3.39E-03

VKORC1 rs9923231 AA	0.88%	-	-31.481	1.03E-03

CYP2C9*2 rs1799853 AG	4.09%	-	-2.66	0.593

CYP2C9*2 rs1799853 GG	95.91%	-	-	-

CYP2C9*3 rs1057910 GT	2.34%	-	-14.91	0.0176

CYP2C9*3 rs1057910 TT	97.66%	-	-	-

Principal Component 1	-	27.44	-0.00943	0.784

Principal Component 2	-	24.42	-0.00697	0.864

A#m Metabolic Pathway	29.71	7.25	-0.331	0.0135

In this cohort, the A#m pathway score was significantly predictive of warfarin dose (F = 6.175, p= 0.0135) (Table 2). There was an improvement in adjusted r^2 ^of 1.3 percentage points (0.259 to 0.272) with the addition of the weighted metabolic pathway score.

### Metabolic pathway score adds information to IWPC pharmacogenetic dosing equation in a cohort of 302 African Americans

Next, we found that A#m was a statistically significant predictor (F = 5.396, p= 0.0208) of the residual dose unexplained by the current IWPC warfarin pharmacogenetic equation (Table 3) [2]. The addition of the A#m PK Pathway to the existing IWPC dose equation in a cohort of African Americans yields a 1.4 percentage point improvement in dose prediction (0.255 to 0.269).

**Table 3:** IWPC Dose Equation with the addition of A#m pathway score

Covariate	Coefficient	p-value
IWPC Dose Equation	1.00	<2E-16

A#m Metabolic Pathway	-0.2935	0.0208

### Weighting method validation vs. LD-pruning in Cooper et al. data and IWPC African American GWAS

We present a comparison of p-values, number of SNPs included, and adjusted r^2^ values for the weighted method vs. several LD cutoffs in Tables S8 and S9. The best adjusted r^2 ^value in the Cooper et. al. data was achieved at an LD-cutoff of 0.3, with an adjusted r^2 ^of 0.498, compared to an adjusted r^2 ^of 0.480 with the weighted method. The highest adjusted r^2 ^value in the IWPC African American GWAS data was achieved with the weighted method, with an adjusted r^2 ^of 0.272. If LD pruning were to be used on the IWPC African American GWAS data, an LD-cutoff of 0.6 produces the highest adjusted r^2 ^value (0.265) among the various cutoffs. In several cases, choice of LD cutoff influences significance due to information loss, whereas the weighted method produces a significant p-value.

## Discussion

Previous genetic analyses of warfarin response have used single-locus or single-gene based approaches in order to discover candidate variants influencing dose [37,40]. Here, we have developed a method that aggregates SNPs along a biologically important pathway known to affect warfarin dosing -- the enzymes of its metabolic pathway. We focused on metabolic enzymes because of their similar direction of effect on warfarin -- degradation. The metabolism genes were chosen from the PharmGKB database of manually curated drug pathways, which are derived from biological evidence in the literature [39]. For the general application of this method to other drug cases, PharmGKB has manually curated pathways for drugs whose biological actions have been well studied. For application to disease, other resources such as BioCarta and the Pathway Interaction Database may be a useful source of pathways. Here, we show that the aggregate pathway effects significantly contribute to the prediction of warfarin dose, and that this pathway score is not solely driven by CYP2C9, the major warfarin metabolizing enzyme (Additional file 1 Table S3).

Our method is based on the assumption that aggregate effects of the mutational burden of the warfarin metabolizing enzymes have a similar effect and direction of effect on the overall sensitivity to warfarin. This strategy assumes that minor alleles generally lead to a decrease or loss of function. This assumption is supported by a recent systematic review of loss-of-function variants in the human genome, which found loss-of-function variants to consistently occur at lower frequencies [41]. Moreover, our method is robust to a small amount of error (i.e. some of the minor alleles could result in a gain of function and not abolish the overall signal) and was empirically observed to be valid for CYP2C9 [18]. Furthermore, this method relies on using biologically validated pathways for SNP aggregation. Because of this experimental validation, such a method would be less likely to produce false positives.

We avoid the information loss and biases of LD pruning by using a weighting scheme based on LD. This method allows each SNP to contribute independent information without double counting. We found that while LD pruning at a cutoff of 0.3 produced an A#m pathway score with the best adjusted r^2 ^in the Cooper data, the weighted method produced the best adjusted r^2 ^in the African American IWPC GWAS data. LD pruning; however, is biased; one does not know a priori what cutoff will produce the best results, and thus, it becomes necessary to choose a reasonable cutoff based on preference. For instance, the LD cutoff of 0.3, which produced the highest adjusted r^2 ^in the Cooper et al. data would have produced one of the worst adjusted r^2 ^values in the African American IWPC GWAS data. In fact, any cutoff below 0.5 would not have produced a statistically significant A#m pathway score in the African American IWPC GWAS data due to information loss. The weighted method allows one to evaluate the data in an unbiased manner without the need for pruning, eliminating the arbitrary step of LD cutoff selection.

**Figure 4:**
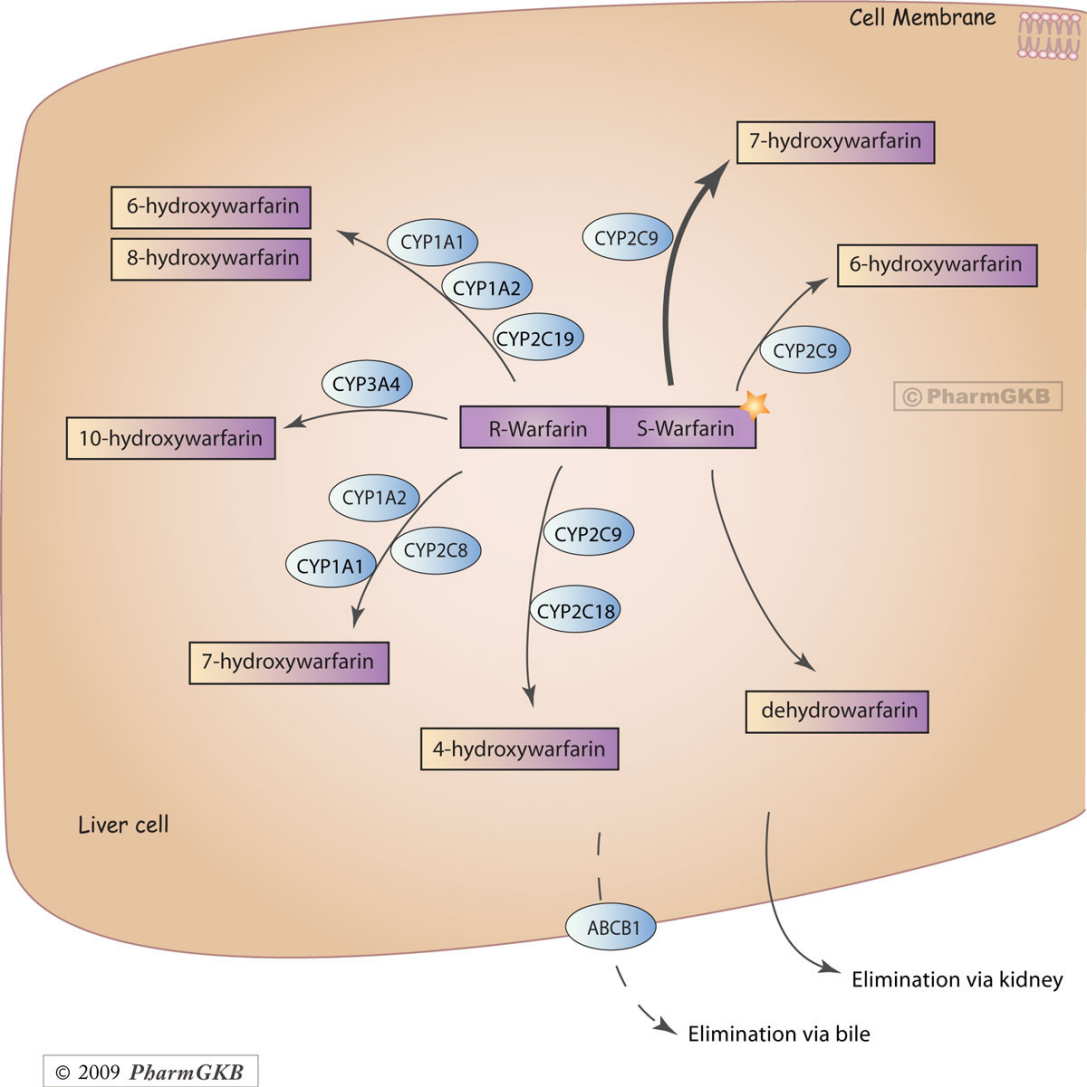
**Warfarin pharmacokinetic pathway**.

In the Cooper et al. data, our A#m pathway score is a statistically significant predictor of dose even when using all available covariate data (including genotype data). Since CYP2C9 has such a large influence on warfarin dose in individuals of European descent, we evaluated whether CYP2C9 was driving the A#m pathway score (Additional file 1 Table S3). Since the CYP2C9 gene score was not statistically significant, we can infer that the significance of the entire pathway is not driven solely by variants in CYP2C9. Furthermore, the remaining pathway score (all metabolic enzymes except CYP2C9) was marginally significant, possibly suggesting that aggregative effects along the rest of the pathway are an important contributor to the significance of the overall pathway. The combinatorial effect of the entire metabolic pathway produces a statistically significant metric for predicting dose -- explaining 1.4 percentage points of the dose. Combinatorial effects in a metabolic pathway make sense from a biological standpoint. As Figure 4 demonstrates, a wide range of enzymes act independently or in concert to metabolize the two warfarin enantiomers. A change in a single enzyme that slows down metabolism may not appear phenotypically, as the other enzymes may compensate or mask the detriment to the overall pathway. However, multiple minor changes in multiple enzymes could create a situation in which the overall pathway is slowed down, in a way that would not have been apparent using single locus or single-gene analysis. The warfarin pharmacodynamic pathway was not a significant predictor of warfarin dose. Biologically, this pathway contains the warfarin target, VKORC1, and upstream/downstream proteins that interact with VKORC1. Many of these proteins have opposite effects on coagulation/warfarin dose -- mutations in CYP4F2 lead to higher warfarin dose, mutations in Protein C and S lead to greater coagulation, while mutations in Factor IX and X lead to under coagulation [42-45]. Because the directionality of each protein's action is not the same (unlike the metabolic enzymes, which all inactivate warfarin or its metabolites in some way), it is not surprising that we find no signal from the combinatorial effects of the pharmacodynamic pathway. Future iterations of the method, which assign directionality to a protein's action, may find signal in this pathway as well.

In the IWPC Malmo cohort, the A#m metabolic pathway score was marginally significant (p = 0.07), suggesting that the pathway score may have an effect on dose prediction in this cohort. The SNPs used in this analysis were different from the SNPs used in the Cooper analysis, due to the use of different genotyping chips. In order to avoid bias, we used all available SNPs in a pathway, which can affect the pathway score if some SNPs do not contribute to the signal and introduce noise. Filtering SNPs based on prior biological knowledge or findings may lead to a stronger signal, but will miss effects of rare variants or SNPs that have not been characterized. Moreover, we expect that the pathway score to be less predictive in populations of European descent compared to populations of African descent because variation along the entire metabolic pathway is lower in populations of European descent [46].

African Americans have greater genetic variation in the cytochrome p450 enzymes making pharmacogenetic warfarin dose prediction particularly difficult. We anticipated that this method would be successful in an African American cohort, where much of the heritability in warfarin dose has not been explained. The addition of the A#m pathway score to the other known covariates for warfarin dose demonstrated that the A#m pathway was a statistically significant contributor to predicting warfarin dose in African Americans. In both the Cooper et. al. and African American analysis, the coefficient for the A#m pathway was negative -- consistent with our assumptions that minor alleles lead to loss of function, since decreased enzyme activity leads to reduced clearance, resulting in a lower therapeutic dose. However, we acknowledge that some alleles may yield a gain of function, and refining our method to take into account the directionality of each SNP's effect would improve our future models.

To show clinical relevance, we calculated the predicted IWPC dose for the African Americans -- this including pharmacogenetic factors such as variants in VKORC1 and CYP2C9. We predicted warfarin dose in the IWPC African American GWAS data using the estimated IWPC dose plus our A#m pathway score, and showed that our A#m pathway score was a statistically significant covariate for dose prediction beyond what is predicted by the IWPC pharmacogenomic algorithm. The pathway score was able to capture 1.4 percentage points of the missing signal in dose prediction, demonstrating that pathway interactions and mutational load may be a contributor to the missing heritability in warfarin dosing.

We used a pathway-based method to show that aggregate effects along biologically important pathways could influence a phenotype (in this case, warfarin dosing). We were able to create an unbiased tool for downweighting SNP contributions based on information similarity (LD), thus eliminating the need for LD pruning prior to aggregation. Currently, we treat each SNP the same - SNPs do not get additional upweighting if functional significance is known. Future methods may assign additional weights to SNPs or genes with biologically known functional relevance that may have a stronger influence on the pathway score. Our method may also have useful predictive value for other phenotypes influenced by pathway-level changes, such as cancer biology.

## Competing interests

RBA is a founder and scientific consultant to http://Personalis.com. JKB has a patent pending for the use of CYP4F2 in warfarin dosing.

## Authors' Contributions

RD contributed to experimental design, pathway and weighting method implementation and statistics, and drafted the manuscript. NPT contributed to experimental design, implementation of the LD-weighting algorithm, and helped draft the manuscript. KJK contributed to experimental design, statistical analysis, and helped draft the manuscript. HS created the A#m scoring metric. SB genotyped and performed quality control on the Malmo Cohort. JKB, TT, YN, MK, MT, NAL, LHC, MP, JAJ, and TEK genotyped and performed quality control on the IWPC African American GWAS data. RBA contributed to experimental design and drafted the manuscript. All authors read and approved manuscript.

## Additional Files

Additional tables can be found in the Supplementary section, in the supplementary file.

## Supplementary Material

Additional file 1 Click here for file

## References

[B1] Pearson TA Manolio TA How to interpret a genome-wide association study JAMA 2008 299 1335 44 10.1001/jama.299.11.1335 18349094

[B2] Klein TE Altman RB Eriksson N Gage BF Kimmel SE Lee MT Limdi NA Page D Roden DM Wagner MJ Caldwell MD Johnson JA Estimation of the warfarin dose with clinical and pharmacogenetic data N Engl J Med 2009 360 753 64 19228618 10.1056/NEJMoa0809329 PMC2722908

[B3] Gage BF Lesko LJ Pharmacogenetics of warfarin: regulatory, scientific, and clinical issues J Thromb Thrombolysis 2008 25 45 51 10.1007/s11239-007-0104-y 17906972

[B4] Kirley K Qato DM Kornfield R Stafford RS Alexander GC National trends in oral anticoagulant use in the United States, 2007 to 2011 Circ Cardiovasc Qual Outcomes 5 615 21 22949490 10.1161/CIRCOUTCOMES.112.967299 PMC3490619

[B5] Wysowski DK Nourjah P Swartz L Bleeding complications with warfarin use: a prevalent adverse effect resulting in regulatory action Arch Intern Med 2007 167 1414 9 10.1001/archinte.167.13.1414 17620536

[B6] D'Andrea G D'Ambrosio RL Di Perna P Chetta M Santacroce R Brancaccio V Grandone E Margaglione M A polymorphism in the VKORC1 gene is associated with an interindividual variability in the dose-anticoagulant effect of warfarin Blood 2005 105 645 9 10.1182/blood-2004-06-2111 15358623

[B7] Johnson JA Gong L Whirl-Carrillo M Gage BF Scott SA Stein CM Anderson JL Kimmel SE Lee MT Pirmohamed M Wadelius M Klein TE Altman RB Clinical Pharmacogenetics Implementation Consortium Guidelines for CYP2C9 and VKORC1 genotypes and warfarin dosing Clin Pharmacol Ther 90 625 9 21900891 10.1038/clpt.2011.185 PMC3187550

[B8] Aithal GP Day CP Kesteven PJ Daly AK Association of polymorphisms in the cytochrome P450 CYP2C9 with warfarin dose requirement and risk of bleeding complications Lancet 1999 353 717 9 10.1016/S0140-6736(98)04474-2 10073515

[B9] Gage BF Eby C Milligan PE Banet GA Duncan JR McLeod HL Use of pharmacogenetics and clinical factors to predict the maintenance dose of warfarin Thromb Haemost 2004 91 87 94 14691573 10.1160/TH03-06-0379

[B10] Gage BF Eby C Johnson JA Deych E Rieder MJ Ridker PM Milligan PE Grice G Lenzini P Rettie AE Aquilante CL Grosso L Marsh S Langaee T Farnett LE Voora D Veenstra DL Glynn RJ Barrett A McLeod HL Use of pharmacogenetic and clinical factors to predict the therapeutic dose of warfarin Clin Pharmacol Ther 2008 84 326 31 10.1038/clpt.2008.10 18305455 PMC2683977

[B11] Sconce EA Khan TI Wynne HA Avery P Monkhouse L King BP Wood P Kesteven P Daly AK Kamali F The impact of CYP2C9 and VKORC1 genetic polymorphism and patient characteristics upon warfarin dose requirements: proposal for a new dosing regimen Blood 2005 106 2329 33 10.1182/blood-2005-03-1108 15947090

[B12] Roper N Storer B Bona R Fang M Validation and comparison of pharmacogenetics-based warfarin dosing algorithms for application of pharmacogenetic testing J Mol Diagn 12 283 91 20228265 10.2353/jmoldx.2010.090110 PMC2860463

[B13] Shaw PB Donovan JL Tran MT Lemon SC Burgwinkle P Gore J Accuracy assessment of pharmacogenetically predictive warfarin dosing algorithms in patients of an academic medical center anticoagulation clinic J Thromb Thrombolysis 30 220 5 20204461 10.1007/s11239-010-0459-3

[B14] Scott SA Khasawneh R Peter I Kornreich R Desnick RJ Combined CYP2C9, VKORC1 and CYP4F2 frequencies among racial and ethnic groups Pharmacogenomics 11 781 91 20504253 10.2217/pgs.10.49 PMC2904527

[B15] Scott SA Jaremko M Lubitz SA Kornreich R Halperin JL Desnick RJ CYP2C9*8 is prevalent among African-Americans: implications for pharmacogenetic dosing Pharmacogenomics 2009 10 1243 55 10.2217/pgs.09.71 19663669 PMC2737687

[B16] Kudzi W Dodoo AN Mills JJ Characterisation of CYP2C8, CYP2C9 and CYP2C19 polymorphisms in a Ghanaian population BMC Med Genet 2009 10 124 19954515 10.1186/1471-2350-10-124 PMC3224726

[B17] Cirulli ET Goldstein DB Uncovering the roles of rare variants in common disease through whole-genome sequencing Nat Rev Genet 11 415 25 20479773 10.1038/nrg2779

[B18] Sagreiya H Berube C Wen A Ramakrishnan R Mir A Hamilton A Altman RB Extending and evaluating a warfarin dosing algorithm that includes CYP4F2 and pooled rare variants of CYP2C9 Pharmacogenet Genomics 20 407 13 20442691 10.1097/FPC.0b013e328338bac2 PMC3098751

[B19] Moore JH Asselbergs FW Williams SM Bioinformatics challenges for genome-wide association studies Bioinformatics 26 445 55 20053841 10.1093/bioinformatics/btp713 PMC2820680

[B20] Cosgun E Limdi NA Duarte CW High-dimensional pharmacogenetic prediction of a continuous trait using machine learning techniques with application to warfarin dose prediction in African Americans Bioinformatics 27 1384 9 21450715 10.1093/bioinformatics/btr159 PMC3087957

[B21] Wang K Li M Hakonarson H Analysing biological pathways in genome-wide association studies Nat Rev Genet 11 843 54 21085203 10.1038/nrg2884

[B22] Walters R Laurin C Lubke GH An integrated approach to reduce the impact of minor allele frequency and linkage disequilibrium on variable importance measures for genome-wide data Bioinformatics 28 2615 23 22847933 10.1093/bioinformatics/bts483 PMC3467741

[B23] Khatri P Sirota M Butte AJ Ten years of pathway analysis: current approaches and outstanding challenges PLoS Comput Biol 8 e1002375 22383865 10.1371/journal.pcbi.1002375 PMC3285573

[B24] Ritchie MD Using prior knowledge and genome-wide association to identify pathways involved in multiple sclerosis Genome Med 2009 1 65 19566917 10.1186/gm65 PMC2703874

[B25] Baranzini SE Galwey NW Wang J Khankhanian P Lindberg R Pelletier D Wu W Uitdehaag BM Kappos L Polman CH Matthews PM Hauser SL Gibson RA Oksenberg JR Barnes MR Pathway and network-based analysis of genome-wide association studies in multiple sclerosis Hum Mol Genet 2009 18 2078 90 10.1093/hmg/ddp120 19286671 PMC2678928

[B26] Torkamani A Topol EJ Schork NJ Pathway analysis of seven common diseases assessed by genome-wide association Genomics 2008 92 265 72 10.1016/j.ygeno.2008.07.011 18722519 PMC2602835

[B27] Fridley BL Batzler A Li L Li F Matimba A Jenkins GD Ji Y Wang L Weinshilboum RM Gene set analysis of purine and pyrimidine antimetabolites cancer therapies Pharmacogenet Genomics 21 701 12 21869733 10.1097/FPC.0b013e32834a48a9 PMC3192305

[B28] Yandell M Huff C Hu H Singleton M Moore B Xing J Jorde LB Reese MG A probabilistic disease-gene finder for personal genomes Genome Res 21 1529 42 21700766 10.1101/gr.123158.111 PMC3166837

[B29] Madsen BE Browning SR A groupwise association test for rare mutations using a weighted sum statistic PLoS Genet 2009 5 e1000384 10.1371/journal.pgen.1000384 19214210 PMC2633048

[B30] Tatonetti NP Dudley JT Sagreiya H Butte AJ Altman RB An integrative method for scoring candidate genes from association studies: application to warfarin dosing BMC Bioinformatics 11 Suppl 9 S9 21044367 10.1186/1471-2105-11-S9-S9 PMC2967750

[B31] Moskvina V Schmidt KM Vedernikov A Owen MJ Craddock N Holmans P O'Donovan MC Permutation-based approaches do not adequately allow for linkage disequilibrium in gene-wide multi-locus association analysis Eur J Hum Genet 20 890 6 22317971 10.1038/ejhg.2012.8 PMC3400741

[B32] Gerstein M Sonnhammer EL Chothia C Volume changes in protein evolution J Mol Biol 1994 236 1067 78 10.1016/0022-2836(94)90012-4 8120887

[B33] Cooper GM Johnson JA Langaee TY Feng H Stanaway IB Schwarz UI Ritchie MD Stein CM Roden DM Smith JD Veenstra DL Rettie AE Rieder MJ A genome-wide scan for common genetic variants with a large influence on warfarin maintenance dose Blood 2008 112 1022 7 10.1182/blood-2008-01-134247 18535201 PMC2515139

[B34] Isma N Svensson PJ Gottsater A Lindblad B Prospective analysis of risk factors and distribution of venous thromboembolism in the population-based Malmo Thrombophilia Study (MATS) Thromb Res 2009 124 663 6 10.1016/j.thromres.2009.04.022 19497611

[B35] Limdi NA Wadelius M Cavallari L Eriksson N Crawford DC Lee MT Chen CH Motsinger-Reif A Sagreiya H Liu N Wu AH Gage BF Jorgensen A Pirmohamed M Shin JG Suarez-Kurtz G Kimmel SE Johnson JA Klein TE Wagner MJ Warfarin pharmacogenetics: a single VKORC1 polymorphism is predictive of dose across 3 racial groups Blood 115 3827 34 20203262 10.1182/blood-2009-12-255992 PMC2865873

[B36] Perera MA Gamazon E Cavallari LH Patel SR Poindexter S Kittles RA Nicolae D Cox NJ The missing association: sequencing-based discovery of novel SNPs in VKORC1 and CYP2C9 that affect warfarin dose in African Americans Clin Pharmacol Ther 89 408 15 21270790 10.1038/clpt.2010.322 PMC3625373

[B37] Cavallari LH Perera M Wadelius M Deloukas P Taube G Patel SR Aquino-Michaels K Viana MA Shapiro NL Nutescu EA Association of the GGCX (CAA)16/17 repeat polymorphism with higher warfarin dose requirements in African Americans Pharmacogenet Genomics 22 152 8 22158446 10.1097/FPC.0b013e32834f288f PMC3261355

[B38] McDonagh EM Whirl-Carrillo M Garten Y Altman RB Klein TE From pharmacogenomic knowledge acquisition to clinical applications: the PharmGKB as a clinical pharmacogenomic biomarker resource Biomark Med 5 795 806 22103613 10.2217/bmm.11.94 PMC3339046

[B39] Whirl-Carrillo M McDonagh EM Hebert JM Gong L Sangkuhl K Thorn CF Altman RB Klein TE Pharmacogenomics knowledge for personalized medicine Clin Pharmacol Ther 92 414 7 22992668 10.1038/clpt.2012.96 PMC3660037

[B40] Liu Y Jeong H Takahashi H Drozda K Patel SR Shapiro NL Nutescu EA Cavallari LH Decreased warfarin clearance associated with the CYP2C9 R150H (*8) polymorphism Clin Pharmacol Ther 91 660 5 22378156 10.1038/clpt.2011.269 PMC3879795

[B41] MacArthur DG Balasubramanian S Frankish A Huang N Morris J Walter K Jostins L Habegger L Pickrell JK Montgomery SB Albers CA Zhang ZD Conrad DF Lunter G Zheng H Ayub Q DePristo MA Banks E Hu M Handsaker RE A systematic survey of loss-of-function variants in human protein-coding genes Science 335 823 8 22344438 10.1126/science.1215040 PMC3299548

[B42] Caldwell MD Awad T Johnson JA Gage BF Falkowski M Gardina P Hubbard J Turpaz Y Langaee TY Eby C King CR Brower A Schmelzer JR Glurich I Vidaillet HJ Yale SH Qi Zhang K Berg RL Burmester JK CYP4F2 genetic variant alters required warfarin dose Blood 2008 111 4106 12 10.1182/blood-2007-11-122010 18250228 PMC2288721

[B43] Feero WG Genetic thrombophilia Prim Care 2004 31 685 709 xi 10.1016/j.pop.2004.04.014 15331254

[B44] Carcao MD The diagnosis and management of congenital hemophilia Semin Thromb Hemost 38 727 34 23011791 10.1055/s-0032-1326786

[B45] Vanden Hoek AL Talbot K Carter IS Vickars L Carter CJ Jackson SC Macgillivray RT Pryzdial EL Coagulation factor X Arg386 specifically affects activation by the intrinsic pathway: a novel patient mutation J Thromb Haemost 10 2613 5 23039000 10.1111/jth.12021

[B46] Limdi NA Arnett DK Goldstein JA Beasley TM McGwin G Adler BK Acton RT Influence of CYP2C9 and VKORC1 on warfarin dose, anticoagulation attainment and maintenance among European-Americans and African-Americans Pharmacogenomics 2008 9 511 26 10.2217/14622416.9.5.511 18466099 PMC2757655

