# Supplementary material for: Pathway analysis of genome-wide data improves warfarin dose prediction
Source: BMC Genomics. 2013 May 28;14(Suppl 3):S11. doi: 10.1186/1471-2164-14-S3-S11 (PMC3829086; doi:10.1186/1471-2164-14-S3-S11)
Supplement: Additional file 1 [file 1471-2164-14-S3-S11-S1.doc]

SUPPLEMENTAL TABLES/FIGURE

| Table S1: Comparing covariates between included and excluded Cooper et. al. data | | |
| --- | --- | --- |
| Covariate | Test Statistic (Chi-squred or Wilcoxon Rank-sum) | p-value |
| Age* | 1034 | 0.0212 |
| Weight | 95.5 | 0.3271 |
| Amiodarone | 0.544 | 0.461 |
| Losartan | 0.0596 | 0.807 |
| VKORC1 status | 0.6943 | 0.7067 |
| CYP2C9 status | 0.7962 | 0.3722 |
| Principal Component 1 | 1508 | 0.9221 |
| Principal Component 2 | 1474 | 0.7979 |
| A#m Metabolic Pathway | 1342 | 0.384 |
| A#m Metabolic Pathway without CYP2C9 | 1277.5 | 0.2417 |
| A#m score for CYP2C9 | 1572 | 0.8447 |
| Dose | 1808 | 0.194 |

The mean and standard deviation of age in the included group was 58.65 and 15.73 and in the excluded group was 66.74 and 16.33.

| Table S2: SNPs of the Metabolic Pathway in the Cooper et. al. data | |
| --- | --- |
| rsid | gene |
| rs1926712 | CYP2C18 |
| rs2860840 | CYP2C18 |
| rs7896133 | CYP2C18 |
| rs1042194 | CYP2C18 |
| rs12243416 | CYP2C18 |
| rs11188059 | CYP2C18 |
| rs1057910 | CYP2C9 |
| rs2475376 | CYP2C9 |
| rs2475377 | CYP2C9 |
| rs4918766 | CYP2C9 |
| rs10509679 | CYP2C9 |
| rs10509680 | CYP2C9 |
| rs9332214 | CYP2C9 |
| rs12251688 | CYP2C9 |
| rs9332169 | CYP2C9 |
| rs4086116 | CYP2C9 |
| rs2253635 | CYP2C9 |
| rs2185570 | CYP2C9 |
| rs4917639 | CYP2C9 |
| rs4646437 | CYP3A4 |
| rs2246709 | CYP3A4 |
| rs1934983 | CYP2C8 |
| rs1934953 | CYP2C8 |
| rs1934956 | CYP2C8 |
| rs6583967 | CYP2C8 |
| rs10509681 | CYP2C8 |
| rs17110453 | CYP2C8 |
| rs11572172 | CYP2C8 |
| rs2185571 | CYP2C8 |
| rs1058932 | CYP2C8 |
| rs7087256 | CYP2C8 |
| rs1891071 | CYP2C8 |
| rs7909236 | CYP2C8 |
| rs11188149 | CYP2C8 |
| rs11572174 | CYP2C8 |
| rs4388808 | CYP2C19 |
| rs10786172 | CYP2C19 |
| rs12767583 | CYP2C19 |
| rs7916649 | CYP2C19 |
| rs1322179 | CYP2C19 |
| rs1322181 | CYP2C19 |
| rs10509678 | CYP2C19 |
| rs4917623 | CYP2C19 |
| rs4646421 | CYP1A1 |
| rs2470893 | CYP1A1 |
| rs2472297 | CYP1A1 |
| rs2472304 | CYP1A2 |
| rs2472299 | CYP1A2 |
| rs2069522 | CYP1A2 |


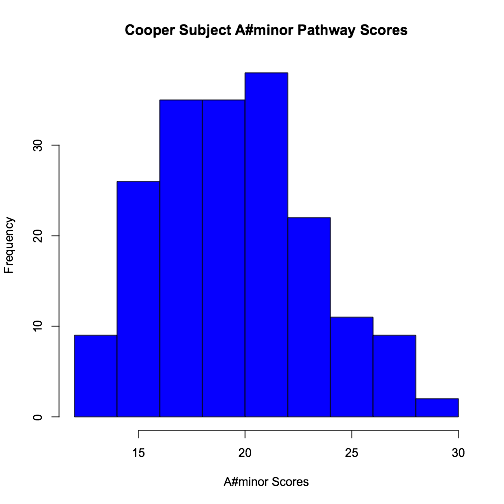


Figure S1: Distribution of A#minor pathway scores in Cooper et. al. data

| Table S3: Pathway Aggregate Effects Without CYP2C9 | | |
| --- | --- | --- |
| Covariate | Coefficient | p-value |
| Age | -0.0407 | 6.61E-05* |
| Weight | 0.0109 | 1.35E-03* |
| Amiodarone | -1.411 | 1.06E-03* |
| Losartan | -0.419 | 4.17E-01 |
| VKORC1 AG | 2.151 | 1.16E-05* |
| VKORC1 GG | 3.816 | 3.50E-13* |
| CYP2C9 *3 homozygote or heterozygote | -1.196 | 1.59E-02* |
| Principal Component 1 | -0.000444 | 9.09E-01 |
| Principal Component 2 | -0.00571 | 3.21E-01 |
| A#m Metabolic Pathway without CYP2C9 | -0.0911 | 6.81E-02 |
| A#m score for CYP2C9 | -0.136 | 1.89E-01 |
| Table S4: IWPC Malmo Cohort patient data, regression coefficients, p-values | | |
| Covariates | Coefficients | p-values |
| Age | -0.304 | 8.03E-06* |
| Weight | 0.209 | 9.33E-04* |
| Height | 0.195 | 6.13E-02 |
| Principal Component 1 | 0.107 | 1.55E-02* |
| Principal Component 2 | 0.024 | 4.63E-01 |
| rs1799853AA CYP2C9*2 homozygote | -8.48 | 0.226 |
| rs1799853AG CYP2C9*2 heterozygote | -8.114 | 1.13E-04 |
| rs1057910CA CYP2C9*3 homozygote | -14.289 | 3.33E-07 |
| rs1057910CC CYP2C9*3 heterozygote | -15.264 | 2.15E-01 |
| rs9923231AG VKORC1 | -8.958 | 1.71E-06 |
| rs9923231AA VKORC1 | -19.76 | 5.76E-13 |
| A#m Metabolic pathway | -0.216 | 7.10E-02 |

| Table S5: SNPs of the Metabolic Pathway in the IWPC Malmo Cohort | |
| --- | --- |
| rsid | gene |
| rs1326832 | CYP2C18 |
| rs1926712 | CYP2C18 |
| rs2281891 | CYP2C18 |
| rs7896133 | CYP2C18 |
| rs11188067 | CYP2C18 |
| rs1409655 | CYP2C18 |
| rs3740367 | CYP2C18 |
| rs2281889 | CYP2C18 |
| rs2281890 | CYP2C18 |
| rs1042194 | CYP2C18 |
| rs12243416 | CYP2C18 |
| rs11188059 | CYP2C18 |
| rs1057910 | CYP2C9 |
| rs1057911 | CYP2C9 |
| rs1799853 | CYP2C9 |
| rs1856908 | CYP2C9 |
| rs2475376 | CYP2C9 |
| rs2475377 | CYP2C9 |
| rs4918766 | CYP2C9 |
| rs9332092 | CYP2C9 |
| rs9332220 | CYP2C9 |
| rs10509679 | CYP2C9 |
| rs10509680 | CYP2C9 |
| rs12772675 | CYP2C9 |
| rs9332214 | CYP2C9 |
| rs1934967 | CYP2C9 |
| rs1934969 | CYP2C9 |
| rs12251688 | CYP2C9 |
| rs9332169 | CYP2C9 |
| rs4086116 | CYP2C9 |
| rs2253635 | CYP2C9 |
| rs2185570 | CYP2C9 |
| rs9332098 | CYP2C9 |
| rs4917639 | CYP2C9 |
| rs11773597 | CYP3A4 |
| rs4646437 | CYP3A4 |
| rs2246709 | CYP3A4 |
| rs3208363 | CYP3A4 |
| rs1341160 | CYP2C8 |
| rs1934983 | CYP2C8 |
| rs1934953 | CYP2C8 |
| rs1934956 | CYP2C8 |
| rs2148551 | CYP2C8 |
| rs6583967 | CYP2C8 |
| rs10509681 | CYP2C8 |
| rs10882520 | CYP2C8 |
| rs11572080 | CYP2C8 |
| rs17110453 | CYP2C8 |
| rs11572172 | CYP2C8 |
| rs2185571 | CYP2C8 |
| rs1058932 | CYP2C8 |
| rs7087256 | CYP2C8 |
| rs1891071 | CYP2C8 |
| rs1058930 | CYP2C8 |
| rs7909236 | CYP2C8 |
| rs2275620 | CYP2C8 |
| rs11188149 | CYP2C8 |
| rs11572174 | CYP2C8 |
| rs4388808 | CYP2C19 |
| rs6583954 | CYP2C19 |
| rs10786172 | CYP2C19 |
| rs12767583 | CYP2C19 |
| rs28399513 | CYP2C19 |
| rs12571421 | CYP2C19 |
| rs7916649 | CYP2C19 |
| rs4304697 | CYP2C19 |
| rs17878459 | CYP2C19 |
| rs1322179 | CYP2C19 |
| rs1322181 | CYP2C19 |
| rs10509678 | CYP2C19 |
| rs4917623 | CYP2C19 |
| rs4646421 | CYP1A1 |
| rs2470893 | CYP1A1 |
| rs1048943 | CYP1A1 |
| rs2472297 | CYP1A1 |
| rs762551 | CYP1A2 |
| rs3743484 | CYP1A2 |
| rs2069526 | CYP1A2 |
| rs2470890 | CYP1A2 |
| rs2472304 | CYP1A2 |
| rs4646427 | CYP1A2 |
| rs4646425 | CYP1A2 |
| rs2472299 | CYP1A2 |
| rs2069522 | CYP1A2 |


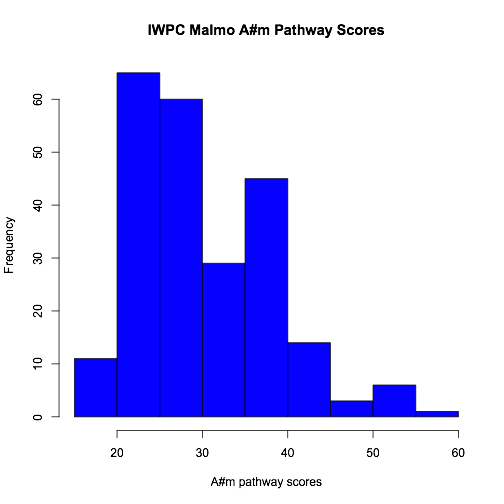


Figure S2: Distribution of A#minor pathway scores in the IWPC Malmo data.


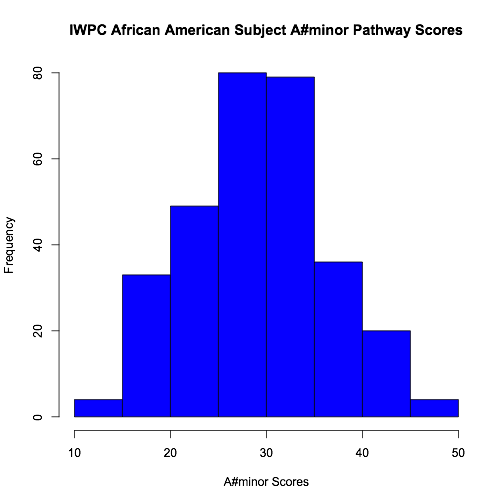


Figure S3: Distribution of A#minor pathway scores in the IWPC African American GWAS Data

| Table S6: SNPs of the Metabolic Pathway in the IWPC African American GWAS Data | |
| --- | --- |
| rsid | gene |
| rs1326832 | CYP2C18 |
| rs1926712 | CYP2C18 |
| rs2281891 | CYP2C18 |
| rs7896133 | CYP2C18 |
| rs11188067 | CYP2C18 |
| rs1409654 | CYP2C18 |
| rs7085394 | CYP2C18 |
| rs1409655 | CYP2C18 |
| rs2296680 | CYP2C18 |
| rs2296681 | CYP2C18 |
| rs1926707 | CYP2C18 |
| rs2296679 | CYP2C18 |
| rs3740367 | CYP2C18 |
| rs2281889 | CYP2C18 |
| rs2281890 | CYP2C18 |
| rs7914753 | CYP2C18 |
| rs2901782 | CYP2C18 |
| rs1010570 | CYP2C18 |
| rs1326830 | CYP2C18 |
| rs1042194 | CYP2C18 |
| rs12243416 | CYP2C18 |
| rs11188059 | CYP2C18 |
| rs1057910 | CYP2C9 |
| rs1057911 | CYP2C9 |
| rs1799853 | CYP2C9 |
| rs1856908 | CYP2C9 |
| rs2475376 | CYP2C9 |
| rs2475377 | CYP2C9 |
| rs4918766 | CYP2C9 |
| rs9332092 | CYP2C9 |
| rs9332220 | CYP2C9 |
| rs10509679 | CYP2C9 |
| rs10509680 | CYP2C9 |
| rs12772675 | CYP2C9 |
| rs17847032 | CYP2C9 |
| rs2256871 | CYP2C9 |
| rs17847036 | CYP2C9 |
| rs9332214 | CYP2C9 |
| rs1934967 | CYP2C9 |
| rs1934969 | CYP2C9 |
| rs12251688 | CYP2C9 |
| rs28371685 | CYP2C9 |
| rs9332169 | CYP2C9 |
| rs4086116 | CYP2C9 |
| rs2253635 | CYP2C9 |
| rs2185570 | CYP2C9 |
| rs12262254 | CYP2C9 |
| rs9332098 | CYP2C9 |
| rs4917639 | CYP2C9 |
| rs12721626 | CYP3A4 |
| rs2737418 | CYP3A4 |
| rs11773597 | CYP3A4 |
| rs4646437 | CYP3A4 |
| rs10250778 | CYP3A4 |
| rs28371759 | CYP3A4 |
| rs28988569 | CYP3A4 |
| rs10270146 | CYP3A4 |
| rs2246709 | CYP3A4 |
| rs3208363 | CYP3A4 |
| rs12721629 | CYP3A4 |
| rs4646440 | CYP3A4 |
| rs1341160 | CYP2C8 |
| rs1934983 | CYP2C8 |
| rs1934953 | CYP2C8 |
| rs1934956 | CYP2C8 |
| rs2148551 | CYP2C8 |
| rs6583967 | CYP2C8 |
| rs10509681 | CYP2C8 |
| rs10882520 | CYP2C8 |
| rs10882526 | CYP2C8 |
| rs11188150 | CYP2C8 |
| rs11572100 | CYP2C8 |
| rs11572102 | CYP2C8 |
| rs11572160 | CYP2C8 |
| rs17110453 | CYP2C8 |
| rs11572162 | CYP2C8 |
| rs11572172 | CYP2C8 |
| rs11572103 | CYP2C8 |
| rs2185571 | CYP2C8 |
| rs1058932 | CYP2C8 |
| rs7087256 | CYP2C8 |
| rs1891071 | CYP2C8 |
| rs1058930 | CYP2C8 |
| rs7909236 | CYP2C8 |
| rs2275620 | CYP2C8 |
| rs11572076 | CYP2C8 |
| rs11188149 | CYP2C8 |
| rs11572174 | CYP2C8 |
| rs4388808 | CYP2C19 |
| rs4986893 | CYP2C19 |
| rs10786172 | CYP2C19 |
| rs12767583 | CYP2C19 |
| rs17878649 | CYP2C19 |
| rs28399504 | CYP2C19 |
| rs28399510 | CYP2C19 |
| rs28399513 | CYP2C19 |
| rs17885857 | CYP2C19 |
| rs12571421 | CYP2C19 |
| rs7916649 | CYP2C19 |
| rs4304697 | CYP2C19 |
| rs17878459 | CYP2C19 |
| rs1322179 | CYP2C19 |
| rs1322181 | CYP2C19 |
| rs10509678 | CYP2C19 |
| rs4917623 | CYP2C19 |
| rs4646421 | CYP1A1 |
| rs4646422 | CYP1A1 |
| rs28399430 | CYP1A1 |
| rs2470893 | CYP1A1 |
| rs4646420 | CYP1A1 |
| rs1048943 | CYP1A1 |
| rs2472297 | CYP1A1 |
| rs762551 | CYP1A2 |
| rs2960193 | CYP1A2 |
| rs3743484 | CYP1A2 |
| rs2069526 | CYP1A2 |
| rs4646427 | CYP1A2 |
| rs12720461 | CYP1A2 |
| rs4646425 | CYP1A2 |
| rs2472299 | CYP1A2 |
| rs17861155 | CYP1A2 |
| rs2069522 | CYP1A2 |

| Table S7: Comparing covariates between included and excluded IWPC African American GWAS data | | |
| --- | --- | --- |
| Covariate | Test Statistic (Chi-squared or Wilcoxon Rank-sum) | p-value |
| Dose | 5105.5 | 0.8172 |
| Age | 5665.5 | 0.5244 |
| Height | 891.5 | 0.4887 |
| Weight | 6117 | 0.3466 |
| Amiodarone | 1.1939 | 0.2745 |
| Aspirin | 0.0363 | 0.8488 |
| VKORC1 rs9923231 AG | 1.3373 | 0.5124 |
| CYP2C9*2 genotypes  rs1799853 | 0.0136 | 0.9071 |
| CYP2C9*3  genotypes  rs1057910 | 0.2352 | 0.6277 |
| Principal Component 1 | 5798 | 0.6811 |
| Principal Component 2 | 6498 | 0.4362 |
| A#m Metabolic Pathway | 7253 | 0.03907 |

For the calculation of the IWPC pharmacogenetic equation dose, we used all clinical and genetic covariates, including the presence of other CYP2C9 variants, if that information was available. Otherwise, the CYP2C9 haplotype status was determined by using the genotypes at rs1799853 (CYP2C9 *2) and rs1057910 (CYP2C9 *3).

| Table S8: Cooper et. al. Weighted vs. LD-pruning A#m pathway p-values | | | |
| --- | --- | --- | --- |
| LD-pruning cutoff | Number of SNPs | A#m Pathway p-value | Adjusted R-squared |
| 0.1 | 7 | 0.0202 | 0.481 |
| 0.2 | 12 | 0.0628 | 0.474 |
| 0.3 | 18 | 0.00135 | 0.498 |
| 0.4 | 21 | 0.00211 | 0.495 |
| 0.5 | 24 | 0.00391 | 0.491 |
| 0.6 | 25 | 0.00857 | 0.486 |
| 0.7 | 27 | 0.0352 | 0.478 |
| 0.8 | 29 | 0.0167 | 0.482 |
| 0.9 | 35 | 0.00253 | 0.482 |
| Weighted Method | 49 | 0.0244 | 0.480 |

| Table S9: IWPC African American GWAS Weighted vs. LD-pruning A#m pathway p-values | | | |
| --- | --- | --- | --- |
| LD-pruning cutoff | Number of SNPs | A#m Pathway p-value | Adjusted R-squared |
| 0.1 | 29 | 0.980 | 0.232 |
| 0.2 | 43 | 0.887 | 0.232 |
| 0.3 | 49 | 0.563 | 0.233 |
| 0.4 | 56 | 0.0803 | 0.240 |
| 0.5 | 60 | 0.000978 | 0.260 |
| 0.6 | 64 | 0.000349 | 0.265 |
| 0.7 | 66 | 0.00323 | 0.255 |
| 0.8 | 71 | 0.006374 | 0.251 |
| 0.9 | 77 | 0.00900 | 0.250 |
| Weighted Method | 122 | 0.0135 | 0.272 |
